# Plasma proteomics reveals white matter lesions in Chinese middle‐aged and elderly people with vascular risk factors

**DOI:** 10.1002/alz70856_101588

**Published:** 2025-12-24

**Authors:** Sisi Peng, Junjian Zhang

**Affiliations:** ^1^ Zhongnan Hospital of Wuhan University, Wuhan, Hubei, China; ^2^ Zhongnan Hospital, Wuhan University, Wuhan, China

## Abstract

**Background:**

White matter lesions (WMLs) are common neuroimaging findings in middle‐aged and elderly populations, often linked to vascular risk factors. However, the burden of WMLs varies widely even among individuals with similar risk profiles, and their molecular mechanisms are not well understood. Recent advances in plasma proteomics enable the non‐invasive identification of biomarkers associated with WMLs. By integrating proteomic data with clinical indicators using machine learning, this study aims to develop a molecular model to assess WML severity and explore its mechanisms.

**Methods:**

This study included two cohorts. In Cohort 1 (*n* = 181), participants were classified into mild (Fazekas < 2, *n* = 97) and severe (Fazekas ≥ 2, *n* = 84) WML groups based on brain MRI. Clinical data, cognitive assessments, and plasma samples were collected. Proteomic profiling was performed using HPLC‐MS/MS to identify differentially expressed proteins. Functional annotation and pathway analyses were conducted with DO, GO, and KEGG databases. Key proteins were selected using LASSO regression, and a predictive model was developed and validated internally. Cohort 2 (*n* = 78) was used for external validation to assess the model's stability and accuracy.

**Results:**

The severe WML group showed significantly worse cognitive performance than the mild group, with lower scores on the MMSE, SDMT, CTT‐Trail 1, and CTT‐Trail 2. Proteomic analysis identified 123 differentially expressed plasma proteins (fold change ≥ 1.5), including 89 upregulated and 34 downregulated. Bioinformatics analysis linked these proteins to vascular diseases, metabolic disorders, and brain injuries, with enrichment in processes like glucagon signaling and fatty acid metabolism. A clinical prediction model incorporating four key plasma proteins (ABI1, ASS1, CXCL12, and EIF2S1) and cognitive scores (DSMT, MMSE, CTT‐Trail 1) demonstrated strong discriminatory performance, achieving an AUC of 0.845 (95% CI: 0.789–0.902) in internal validation (Cohort 1) and 0.840 (95% CI: 0.746–0.934) in external validation (Cohort 2).

**Conclusion:**

This study developed and validated a plasma proteomics‐based model to assess WML severity, integrating key proteins and cognitive scores. The model showed high sensitivity, specificity, and robustness across cohorts, providing a reliable, non‐invasive tool for WML evaluation. These findings offer insights into WML mechanisms and support individualized diagnosis and targeted therapies.

